# Everybody's Page

**Published:** 1888-03-17

**Authors:** 


### Everybody's Page.

Workrooms, in addition to being carefully ventilated, should have plenty of light from the sun.

Pliny says the Gauls invented the art of making soap, and that the Romans acquired it from the Gauls, and introduced it into Italy. Soap factories are known to have existed in Spain in the eighth century; in France about the thirteenth century; and in Great Britain about the fourteenth century.

If the lungs are in a thoroughly sound state they will almost certainly throw off the germs of disease which are present in the air, and are being constantly drawn in by the breath. They reach the lungs indeed, but they find no lodgment there; but if the lung tissues are weakened by the action of some slight disorder, disease germs are apt to find a suitable resting-place, where they may grow and lead to serious mischief.

A sumptuary law, emanating, not from Parliament, as in days of yore, but from those autocrats who rule the world of millinery, has been issued. It is to the effect that birds shall no longer form part of the trimming of hats and bonnets. Birds of paradise and humming-birds, the jewels of the animal kingdom, have become so rare that if the demands of London and Paris are satisfied for a few years more, these demands will have to cease for ever, because there will be no possibility of satisfying them. To protect, therefore, all that remains of the loveliest species of the bird creation, the edict, more terrible than penal servitude to the average woman, has gone forth that she who wears the body of any bird as a part of her dress shall be deemed old-fashioned and provincial.

Some people are really martyrs to principle, and to principle of a very unreasoning kind. They know that exercise is good, and so take conscientiously a walk of a fixed number of miles per diem. "Other people do it and are the better for it; why should it not suit me?" they say, forgetting entirely that "other people" have been used to much open-air exercise from childhood, or perhaps have no daily work that involves strain on mind or body. People whose duties demand any considerable amount of mental or nervous strain often profit more by spending their leisure time in perfect rest than in varying their regular work by another sort of labour which they call exercise. Fresh air and a moderate amount of exertion are very necessary for the maintenance of health; but one may have too much of the former, and fatigue is not always the road to health.

Pianists are not generally looked upon as hard-working people. The world at large regards as work only labour which soils and roughens the hand; all else is only a kind of play. A little calculation of the exertion involved in piano-playing, such as Sir James Paget has made and confided to the students of the London Society for the extension of University Teaching, will dispel this idea. Mademoiselle Janotha, in playing a presto of Mendelssohn's, struck 5,995 notes in four minutes and three seconds. Every one of the notes struck involved two motions, and many of them involved repeated motions of the wrist laterally up and down, and the motion of the elbow and the arm. That would be about twenty-four notes per second, each note involving three distinct muscular motions, or seventy-two motions in every second. For every second of time there were two hundred transmissions of nerve-force from and to the brain. Add to this the emotional strain of putting true expression into the music, and a little reflection will convince us that such work is something more than child's-play.

The commonest of mispronunciations in London is the use of the long *i* for long *a*. Thus one gutter-child will say to another, "Come and *pli* with me," instead of "Come and *play* with me;" and "Take care of the *biby*," instead of of "Take care of the *baby*." It is difficult to say whence this accent arises, for it is not due to any structural defect.

As soon as the crown of a tooth is completed, and long before the root is perfect, the process takes place by which the tooth is "cut," as it is termed. This occurs, not by the tooth forcing or tearing a way or passage for itself through the gum and other tissues, but by these tissues themselves making a way for the tooth's escape.

The more sensitive parts of the body, such as the lips and tongue, can distinguish as interrupted the impulses of a tuning-fork vibrating 1,000 times in a second; and the ear can distinguish interruptions recurring 100 times in a second. But the eye retains the impression of light for a second or more; hence the appearance of a circle of fire when a glowing ember is whirled round by the hand.

Fox, in his "Acts and Monuments," mentions that the "Tartarians," after having vanquished the army of Henry, Prince of Polonia and Siberia, re-counted their victory by taking one ear of every one of the Christians that were slain, and found the slaughter so great that they filled nine great sacks full of ears. Human ears have often been put to strange uses, but surely this, of forming the statistics of a census of the dead, is one of the strangest.

In these days, when machinery and schooling have done their best to make our girls forget what their grandmothers would have described as "the use of their hands," it is pleasant to hear of an exhibition of plain needle-work. The exhibition, which is held at 24, Grosvenor Square, was opened on Monday by H.R.H. Princess Christian, and contains as many as six thousand specimens of needlework, all done by National School girls.

A writer in the *Lady's Pictorial* suggests a new form for charity to take. Reflecting on the numberless injuries to children from burning that our newspapers record, she suggests the formation of a fund for the purchase of fire-guards, to be distributed among poor mothers who are often compelled to leave their children to their own devices for hours at a time. The notion is a kindly one, but we doubt if its utility would not be counteracted by the wonderfully inventive restlessness of childhood. Children who want to play with fire have energy and ingenuity enough to reach their fascinating enemy in spite of either guards or guardians.

While the thoughts of all were concentrated on a life that had passed by a score of years the age allotted to man, Dr. Burney Yeo's remarks on centenarians in the *Nineteeth Century* came very appropriately. Who are those of the human race most likely to attain an age of more than a hundred years? Dr. Yeo's statistics indicate that the essentials for long life are short stature, spare habit, and a good digestion. Although dyspeptics have occasionally lived to great ages, we cannot but feel that to have "good digestion wait on appetite" is no mean weapon in fighting the king of terrors. Other aids to longevity are a life that makes no great strain on the nervous system, and temperance, touching on abstemiousness, in eating and drinking. Moreover, among male centenarians, bachelors were in the minority; but among women, wives, widows, and spinsters were nearly equal in number.

